# Impact of ZrO_2_ Content on the Formation of Sr-Enriched Phosphates in Al_2_O_3_/ZrO_2_ Nanocomposites for Bone Tissue Engineering

**DOI:** 10.3390/ma17081893

**Published:** 2024-04-19

**Authors:** Fabio Caixeta Nunes, Sarah Ingrid Pinto Santos, Luiz Alberto Colnago, Peter Hammer, Julieta Adriana Ferreira, Carlos Eduardo Ambrósio, Eliria Maria Jesus Agnolon Pallone

**Affiliations:** 1 Postgraduate Programme in Materials Science and Engineering, Faculty of Animal Science and Food Engineering (FZEA), University of São Paulo (USP), Av. Duque de Caxias Norte, 225, Pirassununga 13635-900, SP, Brazil; eliria@usp.br; 2 Department of Veterinary Medicine, Faculty of Animal Science and Food Engineering (FZEA), University of São Paulo (USP), Pirassununga 13635-900, SP, Brazil; sarahingrid@usp.br (S.I.P.S.); ceambrosio@usp.br (C.E.A.); 3 Brazilian Agricultural Research Corporation, EMBRAPA Instrumentation, Rua Quinze de Novembro, 1500/1501, São Carlos 13561-206, SP, Brazil; luiz.colnago@embrapa.br; 4 Institute of Chemistry, São Paulo State University (UNESP), Araraquara 14800-900, SP, Brazil; peter.hammer@unesp.br; 5 Fundação Hermínio Ometto, Fundação Hermínio Ometto (FHO), Av. Dr. Maximiliano Baruto, 500, Araras 13607-339, SP, Brazil; julieta.ferreira@fho.edu.br; 6 Department of Biosystem Engineering, Faculty of Animal Science and Food Engineering (FZEA), University of São Paulo (USP), Pirassununga 13635-900, SP, Brazil

**Keywords:** alumina-zirconia, biomaterial, calcium phosphates, surface treatment

## Abstract

This study investigates the profound impact of the ZrO_2_ inclusion volume on the characteristics of Al_2_O_3_/ZrO_2_ nanocomposites, particularly influencing the formation of calcium phosphates on the surface. This research, aimed at advancing tissue engineering, prepared nanocomposites with 5, 10, and 15 vol% ZrO_2_, subjecting them to chemical surface treatment for enhanced calcium phosphate deposition sites. Biomimetic coating with Sr-enriched simulated body fluid (SBF) further enhanced the bioactivity of nanocomposites. While the ZrO_2_ concentration heightened the oxygen availability on nanocomposite surfaces, the quantity of Sr-containing phosphate was comparatively less influenced than the formation of calcium phosphate phases. Notably, the coated nanocomposites exhibited a high cell viability and no toxicity, signifying their potential in bone tissue engineering. Overall, these findings contribute to the development of regenerative biomaterials, holding promise for enhancing bone regeneration therapies.

## 1. Introduction

The utilization of synthetic biomaterials for biomedical applications presents a potential alternative, particularly in scenarios involving a substantial increase in bone tissue lesions and implants [1,2]. Ceramic nanocomposites, for example, are ceramic materials that incorporate nanoscale reinforcements, such as nanoparticles or nanofibers dispersed in a matrix. This joining is responsible for some property improvements, which can increase the mechanical resistance, thermal stability, bioactivity, and other desirable properties. Typically, these nanocomposites are applied in a wide range of applications, such as structural materials, energy systems, electronics, biomaterials, and others [3].

Several processing techniques are employed to obtain nanostructured alumina oxide-based nanocomposites, including traditional methods such as sol–gel, chemical vapor deposition, precipitation, and mixing oxides using ball milling, among others. Currently, advancements in the field of nanotechnology involve various processing technologies, such as the fabrication of functional materials using galvanostatic anodic oxidation, atomic layer deposition, electrospinning, and plasma processing, among others [4,5,6,7]. Al_2_O_3_/ZrO_2_ nanocomposites, for example, have a high mechanical and wear resistance when compared to other ceramic biomaterials [8,9,10]. However, these materials interact minimally with host tissues due to their bioinert nature [11,12,13].

Combining Al_2_O_3_/ZrO_2_ nanocomposites with bioactive materials aims to enhance the biological activity of these materials. These combinations use surface activation methods such as biomimetic coating [14], a method that consists of immersing a substrate in simulated body fluid (SBF) [14,15,16,17]. Incubation enables the formation of calcium phosphate phases of biological interest that are deposited on the bioinert surface of the nanocomposite, increasing the bioactivity of the system [18,19]. This enhancement in bioactivity may render the material more susceptible to interacting with the surrounding biological environment, thereby improving the response in relation to tissue formation [19].

Calcium phosphates are bioceramics that resemble the inorganic components of bones [20,21]. Calcium phosphate phases exhibit distinct degradation rates and solubility, enabling greater adaptability with the desired application for these materials [22,23,24]. The biological performance of these calcium phosphates phases stems especially from the structural arrangement of the ions of each phase governing the physicochemical properties of the phosphates [21,25]. Thus, the bioactivity of calcium phosphates is based on their crystallinity, stability, solubility, and mechanical properties [21,25,26].

A strategy to improve the physical and biological characteristics of calcium phosphates is cationic ion incorporation [27]. Strontium ions (Sr^2+^), for example, are common dopants in calcium phosphates, modifying their solubility, inducing osteoblast proliferation, inhibiting osteoclast reabsorption, and increasing their biological activity [28,29,30]. Therefore, enriching the SBF solution is a viable method for incorporating Sr^2+^ ions during incubation, stimulating the replacement of Ca^2+^ by Sr^2+^ in the phosphate structure [31,32].

Modifications to surface properties are crucial for enhancing the interaction between the bioactive layer of phosphates to be deposited during biomimetic coating and the surface of the nanocomposite [18,33]. These modifications, encompassing surface energy, roughness, wettability, and surface area, among others, are also significantly influenced by the composition of the ceramic substrate [34,35,36,37,38].

Al_2_O_3_/ZrO_2_ nanocomposites, in particular, can be influenced by the quantity of nanometric inclusions of ZrO_2_ in the Al_2_O_3_ matrix, affecting their mechanical, thermal, and electrical properties [8,9]. Furthermore, the addition of ZrO_2_ may induce the formation of calcium phosphates and Sr-containing phosphates [18,33,34]. Despite the potential of Al_2_O_3_/ZrO_2_ nanocomposites, limited research has addressed the influence of varying ZrO_2_ concentrations on phosphate formation, particularly in nanocomposites. Therefore, the main goal of this study is to investigate the effect of ZrO_2_ concentrations on the formation of calcium and strontium phosphates on the surfaces of Al_2_O_3_/ZrO_2_ nanocomposites, aiming to enhance their bioactivity for bone tissue regeneration applications.

## 2. Materials and Methods

### 2.1. Chemical

Nanocomposites were prepared using commercial powders of Al_2_O_3_ AKP-53 (99.99% purity, Sumitomo Chemical, Tokio, Japan) and ZrO_2_ stabilized with 3.0% of yttrium mol (99.99% purity, Nanostructured Materials Inc., Katy, TX, USA).

### 2.2. Obtention of Nanocomposites and Surface Treatment

To prepare the powders, Al_2_O_3_ and ZrO_2_ suspensions were individually prepared in an alcoholic medium using a ball mill. Then, ZrO_2_ suspensions were dripped in 5 (A5Z), 10 (A10Z), and 15% (A15Z) proportions in volume in three agitated Al_2_O_3_ suspensions [18,39]. After drying the three suspensions, the resulting powders were molded into a cylindrical shape of 8 mm in diameter and 4 mm in height using isostatic pressing (200 MPa). Then, the nanocomposites were calcined at 400 °C for 1 h and sintered at 1500 °C for 2 h. The relative density of the nanocomposites was determined using Archimedes’ Principle (ASTM C373-88 2006) as a function of the theoretical density values of Al_2_O_3_ (3.98 g/cm^3^) and ZrO_2_ (5.84 g/cm^3^) [40].

To enhance the wettability and roughness of the nanocomposites, their surfaces were treated with a 5 mol/L phosphoric acid aqueous solution (H_3_PO_4_) for four days in a thermostatic bath at 90 °C [18]. Subsequently, the nanocomposites were rinsed with distilled water and dried at room temperature for 72 h. The treatment with H_3_PO_4_ can influence both wettability and roughness by activating functional sites that promote the deposition of phosphates [24].

### 2.3. Characterizations before Biomimetic Coating

The surfaces of the treated nanocomposites underwent chemical characterization using X-ray photoelectron spectroscopy (XPS) to assess the impact of ZrO_2_ inclusions and their correlation with the coated nanocomposites. XPS analysis was conducted utilizing a commercial spectrometer (UNI-SPECS UHV System) with a pressure lower than 5 × 10^−7^ Pa. The Al Kα line (hν = 1254.6 eV) served as the ionization source, and the analyzer energy was set to 15 eV. Inelastic noise from O 1s, C 1s, Zr 3d, Al 2p, and P 2p wide scan spectra was subtracted using the Shirley method. The composition of the surface layer (<5 nm) was determined based on the relative proportions of spectrum areas, corrected by the Scofield atomic sensitivity factors (±5%). Spectra were deconvoluted using CasaXPS, version 2.3.26, employing a Voigtian function with Gaussian (70%) and Lorentzian (30%) combinations to assign the components of the nanocomposites’ chemical structure in accordance with the National Institute of Standards and Technology (NIST) guidelines [41]. The full width at half maximum (FWHM) ranged between 1.2 and 2.1 eV. Energy scale calibration was achieved using the C 1s hydrocarbon (CC-H) component at 285.0 eV, ensuring a peak position accuracy of ±0.1 eV.

### 2.4. Characterizations after Biomimetic Coating

The nanocomposites were biomimetically coated using a 5× concentrated SBF solution enriched with 1 mmol/L of Sr^2+^ ions [31,42,43]. For this, the nanocomposites were immersed in a solution at 36.5 °C under agitation at 60 rpm for 14 days in an orbital shaker incubator (Marconi, Piracicaba, SP, Brazil) according to the procedure adopted by Barrere et al. (2002) [14]. Solution exchanges and pH measurements were performed every three days (pH meter WTW pH 3210). Then, the nanocomposites were washed with distilled water and dried at room temperature for 72 h.

The calcium phosphates formed on the surfaces of the nanocomposites were identified using X-ray diffraction (XRD) (Rigaku, Tokyo, Japan, model Miniflex 600). A range between 20.0 and 42.5° was chosen as it contains peaks referring to calcium phosphates [19] and strontium [43,44]. To treat the data, the baseline of XRD diffractograms was adjusted using a Savitzky–Golay filter in OriginPro 9.0. The second derivative of each curve was obtained to aid peak deconvolution. Deconvoluted analytical curves were obtained using the Gaussian function (R^2^ > 0.99) in OriginPro 9.0 after normalization using the 35.0° peak (which refers to Al_2_O_3_). Based on the deconvoluted curves, calcium and strontium phosphate phases on the surfaces of the nanocomposites were identified based on Miller indices (*hkl*), according to the Joint Committee for Powder Diffraction database Studies (JCPDS) [45]. The identification of these phases was assisted using Crystallographica Search Match^®^ 3.1.

The phosphate functional groups on the surfaces of the nanocomposites were characterized by Fourier transform infrared absorption spectroscopy (FTIR Spectrometer, Vertex 70 Bruker, Billerica, MA, USA) with a 4 cm^−1^ resolution. The 1000–1650 cm^−1^ range was chosen for its higher incidence of absorption bands attributed to calcium phosphates [46,47,48]. FTIR spectrum baselines were corrected (by the Savitzky–Golay filter) and normalized from a common band between the compositions (653 cm^−1^), attributed to the stretching vibration of Al–O and Zr–O functional groups [35]. Preceding the tests, the coated nanocomposites were subjected to a 24 h drying process at 100 °C to ensure the removal of any excess surface moisture. The semi-quantification of the absolute areas was calculated using the same conditions for all the samples.

### 2.5. Cytotoxicity Assays

This study received approval from the Faculty of Animal Science and Food Engineering, University of São Paulo, according to the ethical committee protocol number: CEUA 5442150419 (ID 001195). The rabbit mesenchymal stem cells (rMSCs) utilized in this research were supplied by the Laboratory of Stem Cells and Gene Therapies at the Faculty of Animal Science and Food Engineering, University of São Paulo.

The cell viability seeded on coated Al_2_O_3_/ZrO_2_ nanocomposites was evaluated using the MTT (3-dimethylthiazol-2,5-diphenyl tetrazolium bromide) assay, an indirect colorimetric method. In this experiment, the MTT assay was performed seeding the cells in the medium containing the nanocomposites. Six samples were used for each condition. The test was adapted from ISO 10993-5 to work with rMSCs [49]. The rMSCs were cultured in a DMEM medium supplemented with 10% fetal bovine serum, 1% L-glutamine, 1% penicillin/streptomycin, 1% MEM nonessential amino acids, and 0.4% amphotericin B in a humidified incubator at 5% CO_2_ and 37.5 °C. A density of 5 × 10^3^ cells was seeded in each well of a 96-well microplate in 100 µL of medium for 24 h before the treatments, except for the control.

Meanwhile, A5Z, A10Z, and A15Z were incubated for 24 h in the medium to be used for the treatment the next day. Control viable cells received the standard medium, which was not incubated with the nanocomposites. After 24 h, each well was incubated for 2 h in 1 mg/mL MTT/PBS. During this time, viable cells produced formazan crystals that were dissolved in dimethylsulfoxide (DMSO) for 5 min, and absorbance was measured at 570 nm in a spectrophotometer (BMG LABTECH, FLUOstar OPTIMA, Saitama, Japan).

### 2.6. Adhesion and Proliferation of rMSCs

To evaluate the interaction between cells and nanocomposites, rMSCs were cultured with the nanocomposites in a 24-well plate at a density of 5 × 10^5^ cells in a DMEM culture medium supplemented as previously described. The culture medium was replaced every 48 h, and cell morphology and proliferation were observed and recorded daily by light microscopy until cells reached 80% confluence. Then, the cell-enriched nanocomposites were fixed in 2.5% glutaraldehyde for 24 h and dehydrated in a range of ethanol concentrations (40, 60, 80, and 100%). The nanocomposites were then dried at room temperature and evaluated via scanning electron microscopy (SEM) (Hitachi, Tokyo, Japan, model TM 3000). At least three samples of each composition were used.

### 2.7. Statistical Analyses

Composition viability was compared using one-way ANOVA analysis (*p* < 0.05), followed by the Dunnett test for multiple comparisons between each treatment and the 100% viability control. Statistical analyses were performed in GraphPad Prism (version 6.0). Pearson correlations were performed in Origin Pro 9.0.

## 3. Results and Discussion

### 3.1. Characterization of Nanocomposites

The nanocomposites A5Z, A10Z, and A15Z showed 97.30 ± 0.15, 98.26 ± 0.37, and 98.33 ± 0.69% (%TD) relative density values, respectively, suggesting that processing led to high particle packing and consolidation [12].

**Figure 1:**
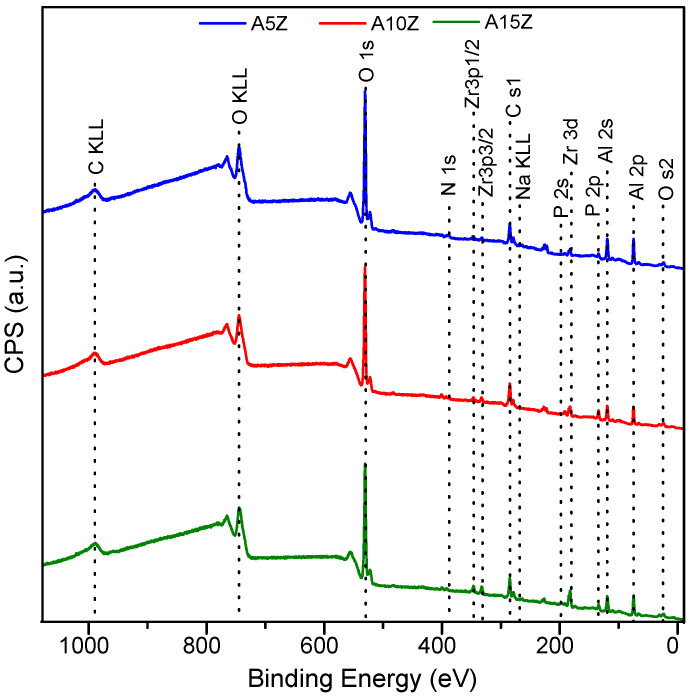
XPS spectra of the treated Al_2_O_3_/ZrO_2_ nanocomposites.

Figure 1 shows the high-resolution XPS exploratory spectra used to determine the atomic concentration (at.%) of the elements on the surface (<5 nm) of the treated nanocomposites (Table 1).

No significant differences can be observed in the positions of spectrum orbital peaks as a function of the increase in ZrO_2_ volume. All compositions contained abundant carbon (C) content due to aliphatic hydrocarbon residues in the environment [50].

**Figure 2:**
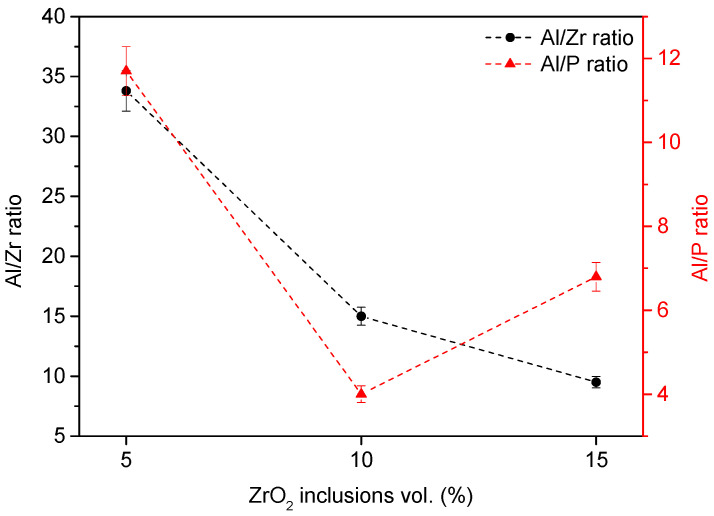
Al/Zr and Al/P ratios of the treated Al_2_O_3_/ZrO_2_ nanocomposites surfaces.

Figure 2 illustrates Al/Zr and Al/P ratios. The Al/Zr ratio expectedly decreased as the ZrO_2_ inclusion volume increased.

Although it was impossible to observe an evident upward or downward trend in Al/P ratios as a function of ZrO_2_ percentages, all compositions contained phosphorus (P) due to the H_3_PO_4_ treatment. The A10Z composition showed a lower Al/P ratio, suggesting significant modification of its surface.

**Figure 3:**
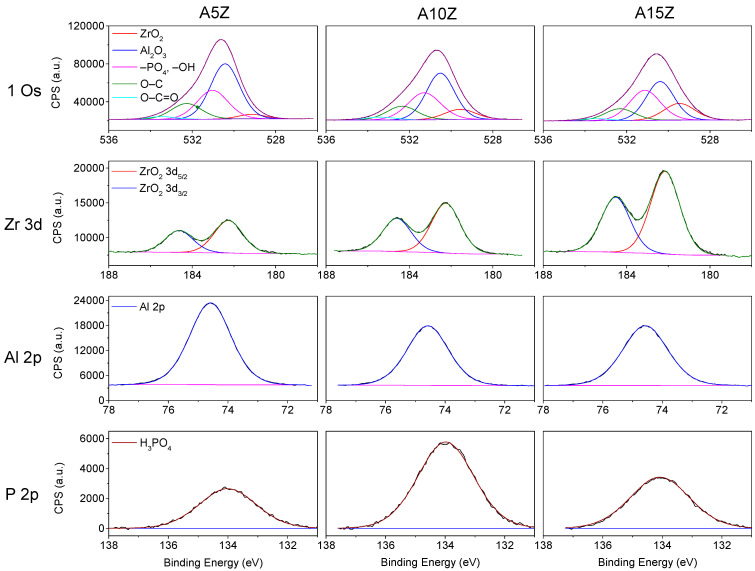
Variations in O 1s, Zr 3d, Al 2p, and P 2p for treated Al_2_O_3_/ZrO_2_ nanocomposite surfaces.

Figure 3 shows the XPS deconvoluted curves.

The nanocomposites had a peak attributed to O 1s of the ZrO_2_ phase at 529.2 eV and a peak attributed to Al_2_O_3_ at 530.5 eV. The –OH and –PO_4_ groups showed the highest binding energy at 531.3 eV and the contaminating oxidized carbon groups refer to O–C at 532.3 eV and O–C=O at 533.5 eV, respectively (Figure 3). Increasing the ZrO_2_ volume slightly reduced the absolute areas of the O 1s orbital (*r_ZrO2 vol/Absolute area O 1s_* = −0.96) and increasing the ZrO_2_ percentage raised the absolute area of –OH and –PO_4_ groups, which may suggest a greater tendency toward calcium phosphate formation after biomimetic coating. Table 2 details the position of the orbital peaks and their respective absolute areas for each composition.

The Zr 3d spectra showed increasing absolute peak areas with higher ZrO_2_ percentages, indicating the influence of ZrO_2_ volume on the surface chemistry. Conversely, the Al 2p spectra exhibited a downward trend in the total absolute area as the ZrO_2_ percentage increased, suggesting that the chemical treatment of the nanocomposite surfaces more significantly impacted ZrO_2_ than Al_2_O_3_.

Overall, these findings suggest that the addition of ZrO_2_ influences the surface chemistry and composition of the nanocomposites, particularly regarding calcium phosphate formation and the distribution of ZrO_2_ and Al_2_O_3_ phases. Further investigations into the implications of these surface modifications on biological interactions, such as cell adhesion and proliferation, would provide valuable insights into the suitability of these nanocomposites for biomedical applications.

### 3.2. Characterization of Nanocomposites after Biomimetic Coating

**Figure 4:**
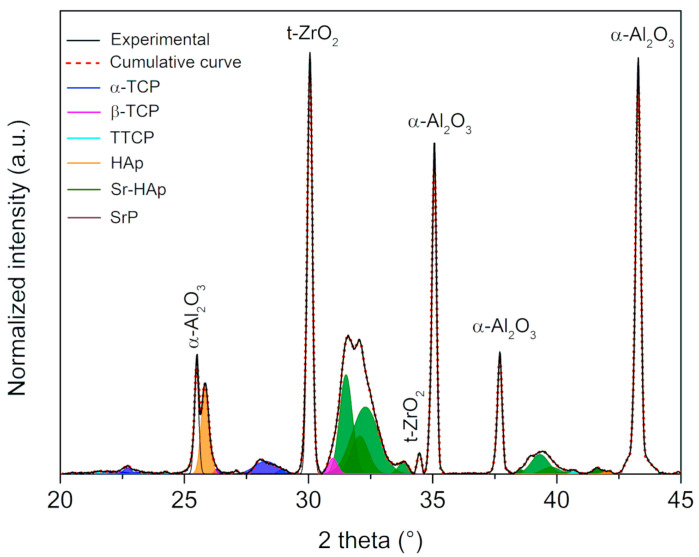
Diffractogram of the A15Z coated surface showing its deconvoluted curves, in which each phosphate phase is shown in a different color.

Figure 4 depicts the deconvoluted XRD curves obtained from the A15Z nanocomposite surface.

The A15Z composition was specifically chosen to exemplify the curves resulting from the deconvolution of the diffractograms, with a consistent methodology applied across all studied conditions. These curves facilitated the identification of phases present on the nanocomposite surfaces following biomimetic coating, with key peaks of each phase pinpointed and phosphate phases characterized based on their profile and position (see Table 3).

Percentage areas were subsequently calculated utilizing the *hkl* diffraction patterns of each phase in accordance with JCPDS standards [45]. Identification of phases on the nanocomposite surfaces, particularly phosphate phases, is crucial for understanding their potential in biomedical applications.

In general, α-tricalcium phosphate (α-TCP) was predominantly assigned to the (202), (151), (132), (241), and (170) planes. Despite not exhibiting a clear correlation with the increase in ZrO_2_ volume, the presence of α-TCP is noteworthy due to its high solubility and osteoconduction capacity [20,25].

Conversely, β-tricalcium phosphate (β-TCP) was identified in the (024), (128), (1010), and (3012) planes, notable for its high resorption rate and thermostability [51,52]. While the A10Z composition displayed a greater abundance of this phase, its percentage decreased significantly with higher volumes of ZrO_2_ inclusion.

The tetracalcium phosphate (TTCP) phase was detected in planes such as (040), (−121), (−151), (232), and (−311), exhibiting a Ca/P ratio of 2.0, higher than other phases, including hydroxyapatite (Ca/P = 1.67). TTCP is biologically relevant due to its bioresorbability and filling capacity [53], with the A5Z composition showing higher percentages of this phase compared to others, potentially enhancing the nanocomposite’s bioactive properties.

Hydroxyapatite (HAp) formation was evident in the (211), (002), (112), (300), and (310) planes, with its formation increasing proportionally with the volume of ZrO_2_ inclusions, displaying a significant Pearson correlation (*r_ZrO2 vol/HAp_* = 0.99). As highlighted by Dorozhkin (2018), the chemical stability in aqueous media and the osteointegration capacity of HAp contribute to its strong chemical affinity with nanocomposite surfaces [22], rendering it bioactive, biocompatible, and osteoconductive [25].

The emergence of strontium phosphate (SrP) and strontium-hydroxyapatite (Sr-HAp) phases indicated the incorporation of Sr^2+^. SrP displayed a slight upward trend with higher ZrO_2_ amounts, observed in planes such as (211), (221), (333), and (312), while Sr-HAp was identified in the (130), (211), (300), and (310) planes. Particularly, HAp formation significantly correlates with ZrO_2_ inclusions, showcasing its potential for enhanced osseointegration and bioactive properties. The incorporation of Sr^2+^ is evident in SrP and Sr-HAp phases, exhibiting varying trends with ZrO_2_ concentration. The highest ZrO_2_ concentration reduces Sr-Hap. Notably, Ca and Sr hydroxyapatites collectively constitute ~60.0%, suggesting promising biomedical application prospects.

**Figure 5:**
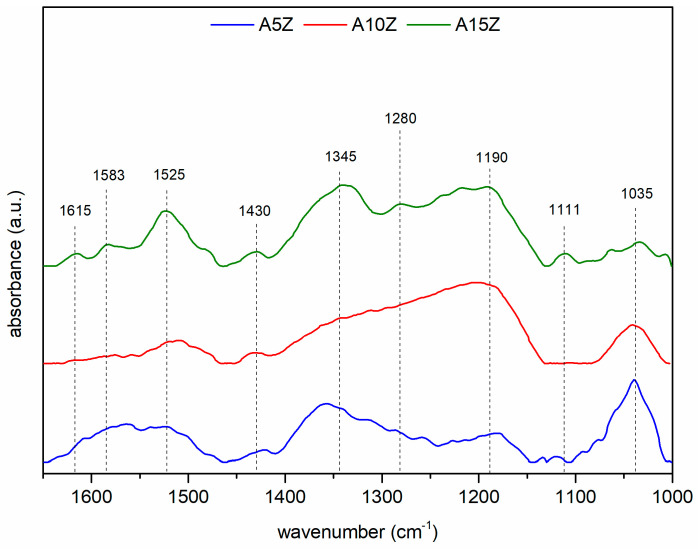
FTIR absorbance spectrum of the coated surfaces.

The FTIR spectra (Figure 5) reveal absorption bands attributed to phosphate functional groups in the HAp phase [48,54], alongside bands associated with α-TCP [55], β-TCP [32,56,57,58], and TTCP phases [55].

The presence of HPO_4_^2−^ [46,59,60], PO_4_^3−^ [61], and CO_3_^2−^ [32,62,63] ions in the spectra suggests their roles as precursors in calcium phosphate formation and their impact on crystallinity and solubility, with higher ZrO_2_ percentages correlating with increased calcium phosphate formation on nanocomposite surfaces, but not necessarily influencing Sr-containing phosphate formation.

**Figure 6:**
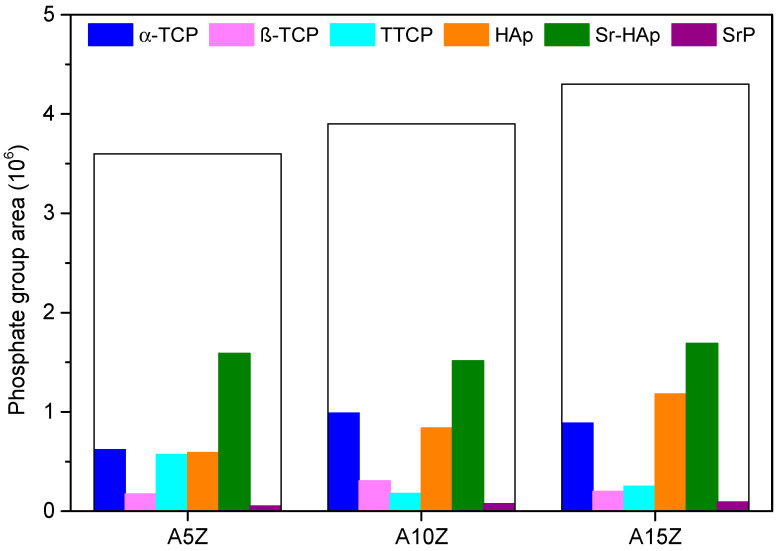
Total and partial area of phosphate phases on coated Al_2_O_3_/ZrO_2_ nanocomposites surfaces.

An analysis of total absolute phosphate areas from the FTIR spectra and the partial areas of each phase from XRD diffractograms in Figure 6 reveals that increasing the volume of inclusions correlates with a higher number of formed phosphates.

It was observed that there are significant correlations between different phosphate phases, such as Sr-HAp and SrP, α-TCP and β-TCP, and Sr-HAp and HAp, suggesting interdependencies in their formation. Despite this, the volume of ZrO_2_ did not significantly influence the formation of phosphate phases containing Sr^2+^. Phosphates exhibit versatility in incorporating various ionic species, including Sr^2+^ ions, which can alter their structural characteristics and biological properties, stimulating biomineralization and bone tissue formation crucial for bone regeneration [29,56].

### 3.3. Cell Viability, Adhesion, and Proliferation of rMSCs

**Figure 7:**
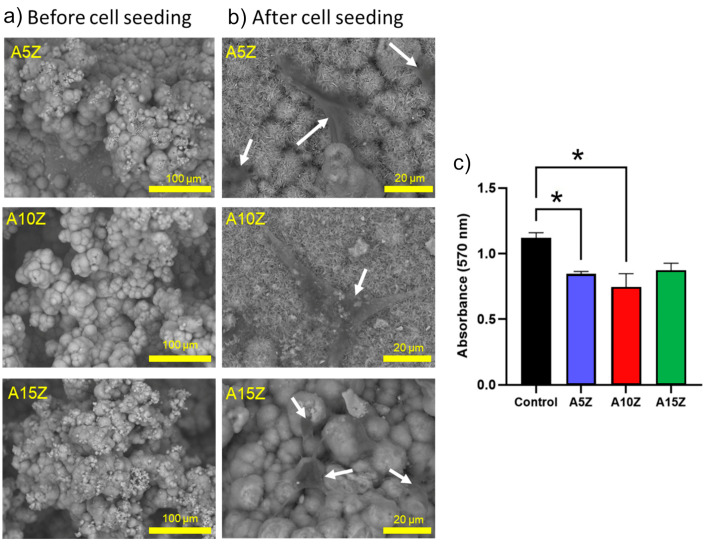
(**a**) SEM analysis without rMSCs, (**b**) SEM analysis with seeded rMSCs, and (**c**) MTT assay (24 h) of coated Al_2_O_3_/ZrO_2_ nanocomposites. * The experimental group compared with the control group, *p* > 0.05.

The morphological analysis in Figure 7 indicates the formation of a bioactive layer on nanocomposite surfaces, influenced by the incorporation of Sr^2+^ ions and potentially affected by factors such as the incubation time, pH, and ionic concentration of the SBF solution [16,43,64].

Regarding the investigation of the biocompatibility of the Al_2_O_3_/ZrO_2_ nanocomposites, non-cytotoxicity was observed after 24 h. All the samples showed no significant differences among them, and the cell viability was above ~70%. Surface modification of the nanocomposites before the biomimetic coating facilitated the deposition of calcium phosphates, which also enhanced cell attachment. The cells demonstrated strong attraction and adhesion to the surface of the nanocomposites. Notably, upon contact with the biomaterial, the cells adopted a fusiform shape with multiple cytoplasmic processes, which interconnected with both neighboring cells and the surface of the biomaterial [64].

It is recognized that further research will be required to comprehensively investigate the adhesion, migration, and morphological changes of the cells, which may be related to their potential osteogenic differentiation. In future studies, our team plans to investigate in detail the dynamic interactions between MSCs and the biomaterial surface, as supported by various studies [65,66,67]. Moreover, as the nanocomposites are dense, the microenvironment of interaction is restricted to the nanocomposite surface. It is expected that further treated and coated nanocomposites with different processing and geometries will be explored, including porous materials. In addition, nanocomposites are presented as an attractive option for the development of novel bone regeneration therapies.

## 4. Conclusions

In conclusion, our study highlights the influence of ZrO_2_ inclusions on the formation of calcium phosphates on Al_2_O_3_/ZrO_2_ nanocomposite surfaces. Varying the quantity of ZrO_2_ inclusions within the Al_2_O_3_ matrix profoundly impacts the nanocomposites’ bioactivity, promoting the formation of different phases of calcium phosphates and Sr-containing calcium phosphates of biological interest. Furthermore, the nanocomposites demonstrated promise for bone tissue engineering, as indicated by their cell viability and bioactivity. Moving forward, further research should optimize ZrO_2_ inclusion levels and explore different shapes to enhance the performance of nanocomposites in bone regeneration therapies.

## Figures and Tables

**Table 1:** Atomic composition (±5%) of the Al_2_O_3_/ZrO_2_ nanocomposites surfaces.

Element	% Atomic Composition (±5%)		
	A5Z	A10Z	A15Z
Oxygen (O 1s)	44.6	43.8	44.3
Carbon (C 1s)	17.7	22.7	19.8
Zirconium (Zr 3d)	1.0	1.7	3.0
Aluminum (Al 2p)	33.8	25.5	28.7
Phosphorus (P 2p)	2.9	6.4	4.2

**Table 2:** Peak positions and absolute areas of O 1s, Zr 3d, Al 2p, and P 2p in treated Al_2_O_3_/ZrO_2_ nanocomposite surfaces.

		A5Z		A10Z		A15Z	
	Assignments	Pos.	Abs. Area	Pos.	Abs. Area	Pos.	Abs. Area
O 1s	ZrO_2_	529.14	5448.7	529.56	14,284.5	529.47	22,754.4
	Al_2_O_3_	530.43	72,827.2	530.52	58,935.4	530.39	48,952.7
	–PO_4_, –OH	531.03	40,612.1	531.29	39,953.0	531.12	41,779.6
	O–C	532.27	21,968.1	532.35	18,900.7	532.32	15,607.3
	O–C=O	533.46	3671.0	533.41	4098.8	533.50	4086.2
Zr 3d	Zr 3d_5/2_	182.26	4639.1	182.25	7236.8	182.15	12,317.5
	Zr 3d_3/2_	184.23	3063.2	184.60	4778.5	184.52	8133.2
Al 2p	Al 2p	74.59	20,052.7	74.58	13,766.2	74.57	15,049.3
P 2p	H_3_PO_4_	134.01	3226.4	133.98	7429.3	134.08	4452.1
C 1s	C–H	284.93	16,721.9	284.90	18,490.9	284.85	16,619.6
	C–O	286.70	2401.2	286.73	4151.9	286.66	3114.0
	O–C=O	288.43	1452.6	288.75	1412.0	288.77	805.2

**Table 3:** Percentage area (%) under XRD diffractogram deconvoluted curves of coated surfaces in the A5Z, A10Z, and A15Z samples and their assignments. Peak values attributed to the substrates were not included for percentage areas.

Phosphates Phases	A5Z	A10Z	A15Z
α-TCP	17.26	25.36	20.64
β-TCP	4.82	7.82	4.64
TTCP	15.81	4.57	5.81
HAp	16.41	21.47	27.46
Sr-HAp	44.21	38.82	39.28
Sr-Phosphate	1.49	1.96	2.17

## Data Availability

Data are contained within the article.
